# Bacterial Community Assembly and Turnover within the Intestines of Developing Zebrafish

**DOI:** 10.1371/journal.pone.0030603

**Published:** 2012-01-19

**Authors:** Qingyun Yan, Christopher J. van der Gast, Yuhe Yu

**Affiliations:** 1 Key Laboratory of Biodiversity and Conservation of Aquatic Organisms, Institute of Hydrobiology, Chinese Academy of Sciences, Wuhan, China; 2 NERC Centre for Ecology and Hydrology, Wallingford, United Kingdom; Charité-University Medicine Berlin, Germany

## Abstract

**Background:**

The majority of animal associated microorganisms are present in digestive tract communities. These intestinal communities arise from selective pressures of the gut habitats as well as host's genotype are regarded as an extra ‘organ’ regulate functions that have not evolved wholly on the host. They are functionally essential in providing nourishment, regulating epithelial development, and influencing immunity in the vertebrate host. As vertebrates are born free of microorganisms, what is poorly understood is how intestinal bacterial communities assemble and develop in conjunction with the development of the host.

**Methodology/Principal Findings:**

Set within an ecological framework, we investigated the bacterial community assembly and turnover within the intestinal habitats of developing zebrafish (from larvae to adult animals). Spatial and temporal species-richness relationships and Mantel and partial Mantel tests revealed that turnover was low and that richness and composition was best predicted by time and not intestinal volume (habitat size) or changes in food diet. We also observed that bacterial communities within the zebrafish intestines were deterministically assembled (reflected by the observed low turnover) switching to stochastic assembly in the later stages of zebrafish development.

**Conclusions/Significance:**

This study is of importance as it provides a novel insight into how intestinal bacterial communities assemble in tandem with the host's development (from early to adult stages). It is our hope that by studying intestinal microbiota of this vertebrate model with such or some more refined approaches in the future could well provide ecological insights for clinical benefit. In addition, this study also adds to our still fledgling knowledge of how spatial and temporal species-richness relationships are shaped and provides further mounting evidence that bacterial community assembly and dynamics are shaped by both deterministic and stochastic considerations.

## Introduction

Animal species have evolved through time in microbial-rich environments where they exist in intimate associations with microorganisms and their communities; which play key roles in the vertebrate host's development [1]. The majority of animal associated microorganisms are present in digestive tract communities, where they, for example: (1) contribute to the host's immune system development; (2) provide digestive capacities/access to nutrients that the host alone could not; (3) stimulate cell renewal in the intestinal epithelium [2]–[6]. Rawls [5] also noted that although studies have demonstrated the importance of gut microbiota in animal biology, the effective translation of such findings to promote human health requires a number of fundamental questions to be addressed. In particular, a key question (and hence the main focus of the current study), is how do microbial communities assemble and develop in conjunction with the development of the host?

Within the current study we used zebrafish (*Danio rerio*) as they possess several unique features that make them an attractive model vertebrate organism to address this question. First, the transparent zebrafish larvae (from time of fertilization through to early adulthood) are a convenient model to perform *in vivo* observations of intestinal bacteria [7]. Second, zebrafish development occurs rapidly and the organization and function of their gut is similar to that of mammals [8]. Third, the whole zebrafish intestine can be easily sampled and measured and is therefore highly convenient for ‘whole-habitat’ microbial analyses at any stage during their lifecycle. The zebrafish intestinal microbiota typically occupies a habitat with definable limits/borders that are comparable to oceanic islands. Therefore, the zebrafish intestine can be considered as an ‘island’ for colonizing bacteria within a ‘sea’ of surrounding (body part) environments [9].

One of the fundamental objectives of ecology is to understand how ecological communities are maintained across spatial and temporal scales. Spatial and temporal patterns of species diversity provide important insights into the underlying mechanisms and processes that regulate biodiversity. One such pattern is the species-area relationship (SAR) which has been used to prioritize conservation efforts particularly in the SLOSS (a single large or several small reserves) debate [10]–[12]. Although spatial scaling of animal and plant species diversity has been well documented over the last century (e.g. [13]–[15]), it is only within the last decade that SARs have started to be addressed at the microbial level (e.g. [9], [16]–[20]). In contrast, the manner in which species richness changes with time has received even less attention in ecology than spatial turnover [15]. Indeed, it is only within the last few years that the temporal analogue of the SAR, the species-time relationship (STR) [14] has been applied at the microbial level [21], [22].

Initially, microbial based studies of SARs and STRs were performed to assess the form of those relationships and how they compared to studies of animal and plant species (see [23], [24]) and more recent microbial SAR and STR based studies have been used to improve our understanding of community assembly and dynamics [20], [22], [25], [26]. Furthermore, SARs and STRs have also been used in an applied context: (1) to distinguish between anthropogenic impacts and underlying natural dynamics [19], [21], [27]; (2) and providing ecological insights for clinical benefit in bacterial infections of cystic fibrosis patient's lungs [28]. As such, the studies cited above emphasize a need to incorporate spatial and temporal scaling into both basic and applied research on microbial species richness patterns. We believe the SAR and STR to be powerful methods for observing spatial and temporal turnover within bacterial communities and especially, as used in the current study, for better understanding community assembly.

Within the present study we investigated patterns of diversity of the intestinal microbiota from individuals of developing zebrafish (from larvae to adult animals [after sexual maturity]) (Table 1) using 16S ribosomal RNA gene-based double-gradient denaturing gradient gel electrophoresis (DG-DGGE). Set within an ecological framework, the overarching aim of the study was to assess bacterial community assembly and development within the developing zebrafish intestinal habitats. Specifically, we assessed how bacterial taxa scaled spatially and temporally (turnover) under increasing zebrafish maturation stages and whether patterns of bacterial diversity were a function of time, increasing habitat (intestinal volume) size, or changing food diet. In addition, we sought to determine to what extent the bacterial community dynamics were driven by either deterministic or stochastic considerations with zebrafish development.

**Table 1:** Zebrafish intestinal volumes (nL) for different individuals sampled at different days post-fertilization (dpf).

	Individual-A	Individual-B	Individual-C	Individual-D
6 dpf	31.26	33.79	29.34	-
11 dpf	16.06	27.76	15.47	-
17 dpf	441.06	310.69	422.29	-
30 dpf	2922.22	4387.85	4468.14	-
45 dpf	3525.28	5661.56	6234.94	-
60 dpf	1609.37	6384.74	6746.82	-
75 dpf	29604.83^a^	12820.73^a^	2926.5^b^	2680.86^b^
90 dpf	13733.44^a^	19407.11^a^	6901.64^b^	3952.30^b^
105 dpf	19569.63^a^	30199.35^a^	6990.61^b^	7881.50^b^

Individuals-A to D denote different replicate zebrafish individuals sampled at a specific dpf.

a Female zebrafish, and

b Male zebrafish.

## Results

### Spatial and temporal species-richness relationships target the zebrafish intestinal bacteria

DG-DGGE of amplified 16S ribosomal RNA (rRNA) gene fragments was used to evaluate the composition of bacteria colonizing the intestines of zebrafish at different stages of development (Table 1 and Table S1). For each sample, the full intestine was removed, photographed, and the diameters and lengths of each fragment were measured to calculate total intestinal volume. The intestinal volumes (habitat sizes) sampled at different stages (from 6 days post-fertilization [dpf] to 105 dpf) varied over three orders of magnitude (ranging from 15.47 to 30199.35 nL, Table 1). Generally, the intestinal volumes of zebrafish increased with developing days (*r* ^2^ = 0.46; *F*_1,28_ = 23.91; *P*<0.0001), although there were occasional outliers to this relationship. These anomalies could be attributed to random sampling and the individual variability may partly arise from developmental differences between individuals. The difference between male and female animals in later stages is another important factor, as female zebrafish are typically larger than males in the adult stages of development (Figure S1).

**Figure 1:**
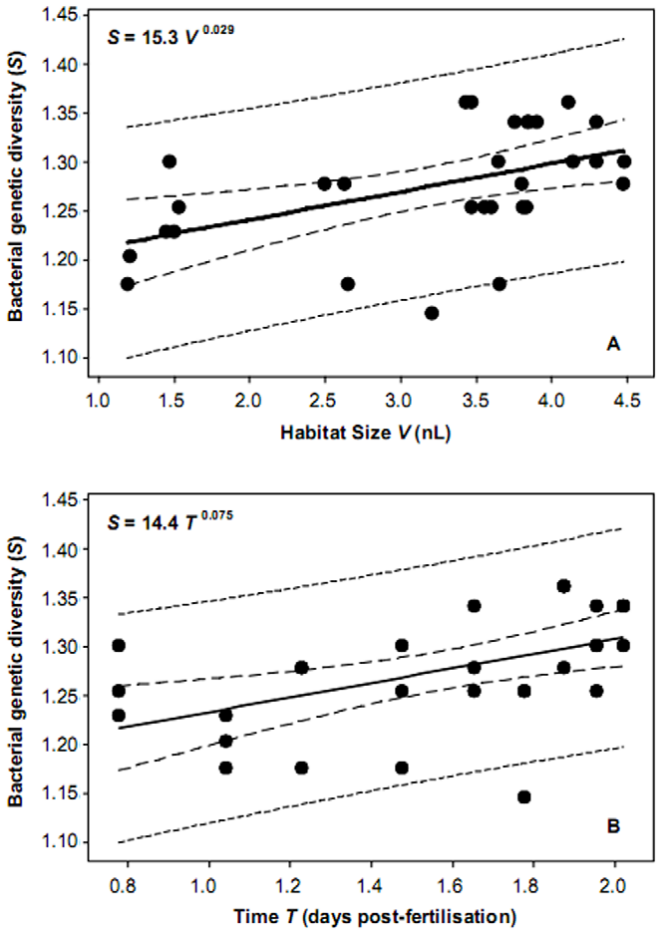
Bacterial richness relationships with (A) habitat size (volume nL) and (B) time (days post-fertilization) plotted on log_10_ scale axes. Given are the (A) species-volume relationship and (B) species-time relationship power law equations. For (A): *r* ^2^ = 0.25, *F*_1,28_ = 9.16, *P*<0.005; and (B) *r* ^2^ = 0.26, *F*_1,28_ = 10.0, *P*<0.004. Also given are the 95% confidence and prediction intervals (inner and outer dashed lines, respectively).

In this study, DG-DGGE band richness (Table S1 and Figure S2) was used to infer the richness of bacterial taxa within each intestine sampled. The species-area relationship was modified to incorporate volume in place of area as the measure of habitat size using the (species-volume relationship, SVR) power law equation, *S* = *cV^z^*. Where *S* is the number of bacterial taxa (DG-DGGE bands) inhabiting an intestine of volume *V*, *c* (the intercept) is an empirically derived taxon- and location-specific constant, and z is the slope of the line (scaling exponent) [16]. Similarly, the species-area relationship power law was modified to describe the relationship between taxa richness and time (dpf), *T* [14]. For clarity, the scaling exponent *z* was changed to *w*, so that the STR power law becomes *S* = *cT^w^* [29]. When both the SVR and STR were plotted, low but comparable spatial and temporal turnover exponents were observed (*z* = 0.029 and *w* = 0.075, Figure 1). Preston [14] proposed that the STR should mimic the SAR (the SVR in this case), following a straight line in log-log space, and would have similar scaling exponents. Although the SVR and STR scaling exponents were different, these differences were small and using the *t*-distribution method [30] the slopes of the SVR and STR were found to be not significantly different (*t* = 1.66; *d.f.* = 1,56; *P* = 0.102). Using this test allowed us to statistically determine that the SVR and STR did indeed have similar scaling exponents as predicted by Preston [14]. As time and volume were covariates, a general linear modelling (GLM) approach was employed to statistically identify whether habitat size (volume, nL), time (dpf), or food diet was the main predictor of bacterial taxa richness (Table S2). In each GLM, time was the only significant predictor, and hence, the observed increase in richness was a function of development time (*r* ^2^ = 0.63; *F*_8,29_ = 4.41; *P*<0.003) and not volume, diet, or any combination of the three potential predictors (Table S2).

### Bacterial community turnover within the intestines of developing zebrafish

Mantel and partial Mantel tests were used to determine which factors (volume, time, and food diet) best predicted community turnover (Table 2). Although differences in volume correlated with community similarity in the Mantel test, when tested again using partial Mantel tests (controlling for the effects of time or food diet) no significant correlations were observed. Differences in food diet did not significantly correlate with community similarity using either Mantel or partial Mantel tests. As observed from the GLM tests, time was the only significant predictor of community turnover, even when controlling for the effects of volume and diet (Table 2).

**Table 2:** Summary statistics for Mantel and partial Mantel tests.

Test type	Test statistic		*P*
Mantel	r(*SV*)	−0.543	0.0001
	r(*ST*)	−0.266	0.0001
	r(*SF*)	0.084	0.082
			
	r(*VT*)	0.357	0.001
	r(*VF*)	−0.037	0.445
	r(*TF*)	0.302	0.0001
			
partial Mantel	r(*SV.T*)	−0.497	0.99
	r(*SV.F*)	−0.542	0.99
			
	r(*ST.V*)	−0.092	0.028
	r(*ST.F*)	−0.307	0.0001
			
	r(*SF.V*)	0.076	0.947
	r(*SF.T*)	0.179	0.99

The Mantel statistic *r*(*AB*) estimates the correlation between two proximity matrices, *A* and *B*. Whereas, the partial Mantel *r*(*AB*.*C*) statistic estimates the correlation between *A* and *B* whilst controlling for the effects of *C*. Also given is *P* to ascertain whether the Mantel and partial Mantel regression coefficients were significantly different from zero following 9,999 permutations. Given are bacterial community similarity *S* (Sørensen index) and also *V*, *T*, and *F*, which are differences in habitat size (volume, nL), temporal distance (days post-fertilization), and food diet, respectively.

**Figure 2:**
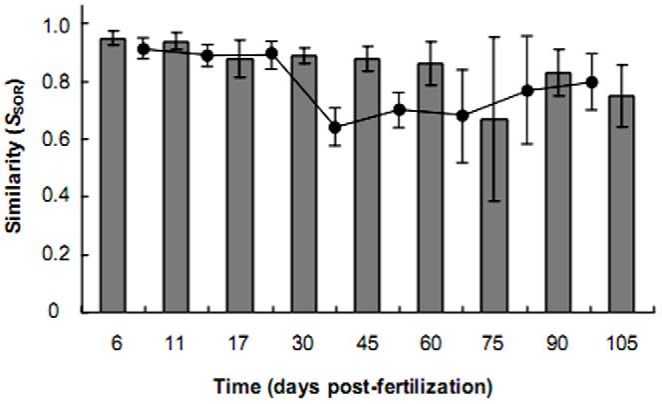
Changes in bacterial community similarity within and between time points. Bars indicate within time point similarity and circles represent similarity between adjacent time points. Similarity is measured by the Sørensen index of similarity (*S*_SOR_). Error bars represent the standard deviation of the mean.

Similarities and differences in the composition of the bacterial communities within and between adjacent stages of development were assayed using the Sørensen index of similarity (Figure 2). Within time point similarity, although highly conserved (0.85±0.09), decreased over the course of the study indicating that bacterial communities within the zebrafish intestines became more divergent with increasing maturation of the hosts. When similarity between adjacent developmental stages was compared, it revealed little change in similarity up to the 30 dpf stage (0.09±0.01). After 30 dpf the rate of change in community composition increased to 0.33±0.06 (Figure 2).

**Figure 3:**
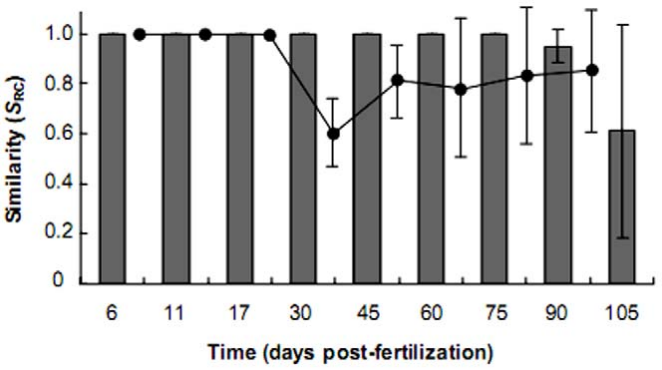
A comparison of changes in similarity values for within and between time points. Bars indicate within time point similarity and circles represent similarity between adjacent time points. Similarity is measured by the Raup and Crick (*S*_RC_) probability-based index of similarity. 0.95>*S*_RC_>0.05 similarity is no greater than expected by chance, *S*_RC_<0.05 significant dissimilarity, *S*_RC_>0.95 significant similarity. Error bars represent the standard deviation of the mean.

To test, to what degree, the bacterial community assembly and dynamics were driven by stochastic (random) or deterministic (niche) considerations, bacterial community profiles were compared using a Monte Carlo procedure to determine whether any two samples were more or less similar than would be expected by chance. Using the Raup and Crick probability-based similarity index (*S*_RC_), the stochasticity and determinism in the data was investigated by recording within and between adjacent developmental stage samples taken pair-wise, whose compositional similarities were not more or less expected by chance (0.95>*S*_RC_>0.05) or significantly similar (*S*_RC_>0.95) or dissimilar (*S*_RC_<0.05). From 6 to 75 dpf within developmental stage *S*_RC_ values remained constant at 1.00±0.00, decreasing to 0.95±0.07 and still significantly similar at 90 dpf, and then less than 0.95 to 0.61±0.43 at 105 dpf (Figure 3). This indicated that within developmental stage similarities were driven by deterministic considerations up to 90 dpf. *S*_RC_ values between adjacent developmental stages up to 30 dpf did not deviate from 1.00±0.00. After 30 dpf to 105 dpf the *S*_RC_ values fell below 0.95 to a mean value of 0.60±0.11. Or rather similarities switched from deterministic to stochastic driven assembly after 30 dpf (Figure 3).

## Discussion

Intestinal microbial communities are functionally essential in providing nourishment, regulating epithelial development, and influencing innate immunity in the vertebrate host [5], [31]. Studies of intestinal microbiota in humans and vertebrate model organisms are revealing a previously unrealised high bacterial diversity and high compositional variability between individuals [32], [33]. It is also established that the bacterial diversity in the intestine is a result of co-evolution between the host and its microbial community [32]. What is still poorly understood is how intestinal bacterial communities assemble. Set within an ecological framework we studied the spatial and temporal scaling of intestinal bacterial communities in zebrafish, to better understand community assembly and dynamics in the development of this well established model vertebrate organism.

Following the ecological definition of an island habitat [9], [15], we considered the zebrafish intestine as an ‘island’ within a ‘sea’ of surrounding environments in similar manner that lakes and ponds are islands within a sea of land. In addition, as vertebrates are born free of microorganisms, the bacterial populations that colonize the intestinal tract must immigrate from outside of the island [32]. Zebrafish are ideal as a model vertebrate organism as they are small, develop quickly, and are easy to rear. All of these advantages make it convenient to sample the whole intestinal niche for analyzing bacterial spatial and temporal patterns of diversity. Also this provides a true ‘whole-habitat’ analyses that is not dependant on taking representative sub-samples which has attracted criticism by some researchers [34] for previous microbial spatial scaling based studies. Here we applied DGGE to analyze the richness and composition of zebrafish microbiota, as it has been used successfully in previous studies of spatial and temporal species richness relationships (e.g. [16], [22], [35]). Although DGGE fingerprinting can't pick up some rare species, DGGE profiles do provide an image of most components of the microbial community free of inventory limitation [22]. To improve the resolution somewhat, we used double-gradient DGGE which further incorporates a gradient in acrylamide concentration [36] to separate the amplified bacterial 16S rRNA gene bands.

The value for the SAR scaling exponent (*z*) has been shown to be consistent across animal and plant species, but differs between islands (0.2 – 0.39) and non-isolated sample areas of contiguous habitat (0.1 – 0.2) [15]. Likewise, a remarkable degree of regularity in the STR has been found were the temporal scaling exponent (*w*) ranged from 0.1 – 0.51 [23]. This has been mirrored, to some degree, for exponents of bacterial spatial and temporal species-richness relationships [23], [24]. In the current study, we found significant species-volume and species-time relationships (Figure 1) which both had similar (to each other) but low turnover exponents when compared to expected values (as briefly detailed above) of *z* and *w*. These analyses indicated that turnover of bacterial taxa within the zebrafish intestines was low. In addition, time was found to be the main predictor of both bacterial richness (Table S2) and community composition (Table 2).

A central question in ecology is why so many species can coexist even when there are so few different apparent niches in a given habitat. Ecologists have attempted to explain this by understanding how communities assemble. This in turn has led to two opposed opinions of deterministic or stochastic assembly. The deterministic view asserts that communities assemble in a defined manner as a result of the number of available niches and competition for those niches in a habitat [37], [38]. Conversely, the stochastic view assumes that assembly is random, making neutral assumptions about individuals within a community, where neutrality is defined as ecological equivalence among all individuals of every species [39]. Put simply, all individuals have the same chance of immigrating into the local community, and reproducing or going extinct once there.

Two previous independent microbial studies have demonstrated that temporal scaling exponent values reflect the degree to which a community is influenced by both deterministic and stochastic considerations where low turnover is indicative of deterministic (niche driven) processes at work. For example, van der Gast and colleagues [22] sampled five bioreactors of fixed volume size along a gradient of increasing industrial wastewater concentrations (0%, 25%, 50%, 75%, and 100%) and consequently decreasing municipal wastewater over a 154 day period. The rationale for the study was to ascertain how bacterial taxa scaled temporally under increasing selective pressure exerted by the wastewater blends in the bioreactor systems, and to what extent bacterial community dynamics were driven by either stochastic or deterministic considerations across the established selective pressure gradient. The authors found that as the industrial wastewater increased in concentration across the bioreactors, a gradual switch from stochastic (random) community assembly to more deterministic (niche) based considerations was observed. This was also reflected in differences in STR scaling exponents (*w*), where *w* decreased as selective pressure (industrial wastewater) increased (0% [*w* = 0.512], 25% [0.432], 50% [0.315], 75% [0.206], and 100% [0.162]) [22]. These results would suggest that as selective pressure and therefore the level of determinism in community assembly increases then temporal turnover of bacterial taxa decreases as a result.

In a recent study, Ayarza and Erijman [25] also indicated that the dynamics of activated sludge bacterial communities are determined by a balance between neutral and deterministic components. However, they used a different line of evidence to demonstrate this. By manipulating the size of the meta- or source community used to form local bioreactor communities they showed, using the goodness of fit to a neutral community model modified for prokaryotes [40], that the higher the number of species in the reservoir from which the local community is drawn, the more important the stochastic component is in the formation of the activated sludge communities. Conversely, when the source community diversity was lower, the more important the deterministic component is in community assembly. To verify this, they also analysed the effect of source community size on the rate of temporal turnover. They found that turnover was greater in bioreactor communities (*w* = 0.31±0.05) formed from more diverse metacommunities when compared to local communities formed from less diverse source communities (*w* = 0.16±0.02) [25]. In short, they found that the larger the metacommunity size the greater the turnover and stochasticity in the dynamics of the local communities.

In the current study, we observed that bacterial communities within the zebrafish intestines were deterministically assembled (reflected in the low STR exponent) switching to stochastic/random assembly in the later stages of zebrafish development (Figure 3). What is indicated from this study and the studies briefly detailed above, is that a continuum of both deterministic and stochastic processes can exist. The wider implications are that purely neutral community assembly models should incorporate the influence of deterministic factors and vice versa as recently performed by Ofiteru et al. [41], who combined niche and neutral effects in a model for ammonia oxidizing bacteria involved in wastewater treatment.

In conclusion, as humans and other vertebrates are essentially born free of microorganisms [32], this study is of importance as it provides a novel insight into how intestinal bacterial communities assemble in tandem with the host's development (from early to adult stages). It is our hope that by studying intestinal microbiota of this vertebrate model using such or some more refined approaches in the future could well provide ecological insights for clinical benefit. In addition, this study also adds to our still fledgling knowledge of how spatial and temporal species-richness relationships are shaped and provides further mounting evidence that bacterial community assembly and dynamics are shaped by both deterministic and stochastic considerations.

## Materials and Methods

### Ethics statement

All experiments involving zebrafish were performed under protocols approved by the Institutional Animal Care and Use Committee of Institute of Hydrobiology, Chinese Academy of Sciences (Approval ID: keshuizhuan 08529).

### Experimental setup and zebrafish (*Danio rerio*) husbandry

Wild-type zebrafish (AB strain) were reared using universal glass aquarium systems under standard laboratory conditions. In brief, fully aerated tap water was used during the experiment, and a stable water temperature (28±0.5°C) as well as 14/10 hours light/dark cycle were strictly controlled to decrease possible effects from environmental perturbation. Zebrafish embryos were obtained from spawning adults as previously described [42]. Normally developed embryos were selected for subsequent experiments and abnormal ones were discarded. A 90% v/v water change was performed each day starting at 3 days post-fertilization (dpf) when larvae hatch from the axenic environment of their protective chorions. The intestine is then colonized by microbes within following 12–24 h [1], [7], by 5 dpf gut morphogenesis has proceeded to a stage that supports feeding and digestion [8], [43], [44]. Beginning at 7 dpf when yolk is completely absorbed [44], feeding zebrafish twice daily (8:00 and 15:00, respectively) with cultured *Paramecium*, from 12 to 15 dpf with 20 µm mesh filtrated boiled egg yolk, and subsequently feeding with brine shrimp. The faeces and leftover food were removed timely to keep a clean environment.

### Intestinal sampling and volume measurement

As zebrafish larvae only resorb the yolk until the gut is fully functional by the 5 dpf [8], [43], [44], the first stage of intestinal sampling was performed at 6 dpf (without any external feeding). Under the breeding conditions applied, the zebrafish embryos developed to have the ability of spawning at the time of 120 dpf. Therefore, the last stage of sampling was conducted at 105 dpf. To investigate zebrafish intestinal bacterial communities at different developmental stages, three individuals were randomly selected ≤60 dpf (each sampling was conducted before feeding), and four individuals were selected from 75 dpf onwards (two males and two females, when body sizes between male and female zebrafish become significantly different, Figure S1). The whole intestine of each selected individual was removed aseptically under a dissecting microscope (see Text S1). The intestinal tract was photographed under microscope using professional software (AxioVision, Zeiss) for subsequent measuring and calculating intestinal volume.

Intestinal volume was used as being indicative of ‘whole-habitat’ size in the present study. Volume was calculated using standard volumetric equation for cylinder as previously described [45]. To compensate for the reduced volume of intestine tapers, sections with different diameters were treated as individual cylinders. Theoretically, intestinal diameters and lengths were measured according to calibrated scale of AxioVision software at sections of the anterior-intestine (AI), middle-intestine (MI), and posterior-intestine (PI) as previously described [46]. When necessary (i.e., visible tapers within segments of AI, MI, or PI), additional measures were added and the intestine was then treated as more than three sections (up to 12 segments for some adult individuals).

### DNA extraction and PCR amplification

Each collected intestinal sample was initially incubated in buffer ASL (provided with Qiagen DNA extraction Kit) at 70°C water bath for 1 minute, and then homogenized by repeated passage through a 20-gauge needle as previously described [8]. Genomic DNA was then extracted using a QIAmp DNA Stool Mini Kit (Qiagen) according to the manufacturer's instructions. To amplify bacterial 16S ribosomal RNA gene (rDNA), PCRs were prepared containing 1×buffer (without MgCl_2_), 2 mM MgCl_2_, 0.05 unit/µl *Taq* DNA polymerase (MBI Fermentas), 80 µM of each deoxynucleotide triphosphate (MBI Fermentas), approximately 1 ng/µl DNA template, and 0.3 µM of each bacterial target primer 357F-GC (*Escherichia coli* 16S rDNA positions 341 to 357) and 518R (*E*. *coli* 16S rDNA positions 518 to 534) [47]. Touchdown PCR was performed on a S1000™ thermal cycler (Bio-Rad) using the following conditions: 5 min at 94°C, followed by 10 cycles of 30 sec at 94°C, 30 sec at 67−58°C (in the first cycle annealing was performed at 67°C, the temperature was then decreased by 1°C each cycle), and 60 sec at 72°C. This procedure was followed by 20 cycles of 30 sec at 94°C, 30 sec at 57°C and 60 sec at 72°C, with a post-amplification extension of 10 min at 72°C. PCR products were visualized using 1.4% agarose gels stained with ethidium bromide. Negative controls were always prepared in the same manner as the samples except that DNA was excluded from the mixture.

### DG-DGGE

Approximately equal amounts of PCR product from each sample were separated on a double gradient polyacrylamide gel. DG-DGGE analysis was performed with a DCode™ system (Bio-Rad) using 7.5–9.0% polyacrylamide gel (acrylamide: bisacrylamide = 37.5: 1) with a 40–50% denaturing gradient. Electrophoresis was performed at 60°C with 100 V for 12 h according to the method described previously [48]. After that, gels were stained in 1×TAE buffer containing 1×SYBR Gold (Molecular Probes) for 20 min, and the gel was photographed using a Gel Doc™ XR imaging system (Bio-Rad). DGGE bands were originally assigned and matched using the Quantity One® software (Bio-Rad, version 4.6.9), and the banding patterns were then carefully checked and revised manually.

### Statistical analyses of data

The number of bands present in a sample (Table S1 and Figure S2) was used to infer estimates of taxa richness as previously described and discussed [20], [22]. The species-volume relationship (SVR) and species-time relationship (STR) were used to visualize and statistically compare differences in bacterial spatial and temporal scaling across the intestines of zebrafish as previously described [16], [22]. A general linear modelling approach (GLM) was used to ascertain what underlying factors shaped bacterial richness patterns [49].

To examine differences in bacterial beta diversity, similarities and differences in community composition were determined using the Sørensen index of similarity (*S*_SOR_) using the PAST program available from the University of Oslo website link (http://folk.uio.no/ohammer/past) run by Øyvind Hammer. In the current study we used both differences in habitat size (volume) and temporal difference between samples taken pair-wise instead of geographical difference. In addition, using Mantel and partial Mantel tests allowed us to determine how patterns of beta diversity are influenced by environmental factors [17]. Mantel and partial Mantel tests were performed as previously described [17] using the program of XLSTAT 2010 (Addinsoft, France).

Band matching data were stored as a binary matrix and analyzed using the Raup and Crick probability-based index of similarity (*S*_RC_) [50]. The *S*_RC_ was chosen as this measure has advantages over non-probability based similarity indexes, such as the Jaccard or Sørensen coefficients, when comparing the diversities of binary data such as DGGE bands [51]. The *S*_RC_ provides a measure of statistically significant similarity and dissimilarity at the 95% confidence level, and is less affected by sampling bias [50], because it can identify those relationships between faunas that are unlikely to be due by chance. Also, an additional feature of the index is that biogeographic data are weighted on the basis of frequency of occurrence, so that widespread taxa do not have a disproportionate influence on measurement of similarity [51]. This method distinguishes similarities arising from chance band matches from those arising from bands matching, at a greater or lesser level, than expected by chance. The index, using Monte Carlo simulations (2000 per pair-wise comparison), evaluates the number of species common to two samples, against the number of species expected to be shared, if two such samples are randomly generated from a pooling of the original samples. The similarity index is the probability that the randomized similarity would be less than or equal to the observed similarity, and *S*_RC_ values above 0.95 or below 0.05 signify differences, which are not random assortments of the same species (bands) [52]. The *S*_RC_ was calculated using the PAST program.

GLMs, regression analyses, coefficients of determination (*r* ^2^), residuals and significance (*P*) were calculated using software of Minitab 15 (Minitab, University Park, PA). The *t*-distribution method was used to compare the regression line slopes as described in Fowler and colleagues [30].

## Supporting Information

Figure S1 **Body weight (g) comparison of the adult individuals.** Asterisks indicate significant difference between female (**♀**) and male (**♂**) zebrafish within stage (two-tailed Student's *t*-test).
(TIF)

Figure S2 **DG-DGGE patterns of the amplified 16S ribosomal RNA (rRNA) gene fragments, each band was considered as an operational taxonomic unit (OTU).** For each sample code, the number represents days post-fertilisation (dpf) and the letter refers to a different zebrafish individual sampled at that dpf (to be continued).
(TIF)

Table S1 DG-DGGE presence-absence profiles for bacterial communities sampled from the intestines of different zebrafish individuals over development time.
(DOC)

Table S2 General linear models and summary statistics.
(DOC)

Text S1 Intestine removal methods.
(DOC)
