# Supplementary figures and images for: Bacterial Community Assembly and Turnover within the Intestines of Developing Zebrafish
Source: PLoS One. 2012 Jan 19;7(1):e30603. doi: 10.1371/journal.pone.0030603 (PMC3261916; doi:10.1371/journal.pone.0030603)

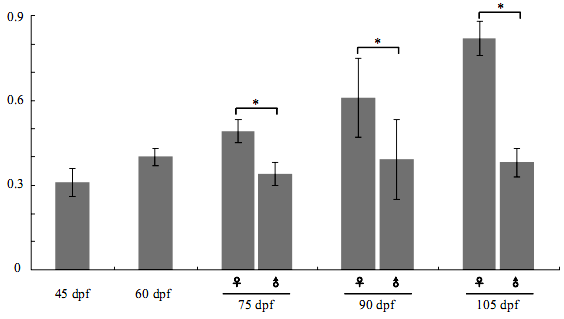

Supplement: Figure S1 — Body weight (g) comparison of the adult individuals. Asterisks indicate significant difference between female (♀) and male (♂) zebrafish within stage (two-tailed Student's t-test). (TIF) [file pone.0030603.s001.tif]

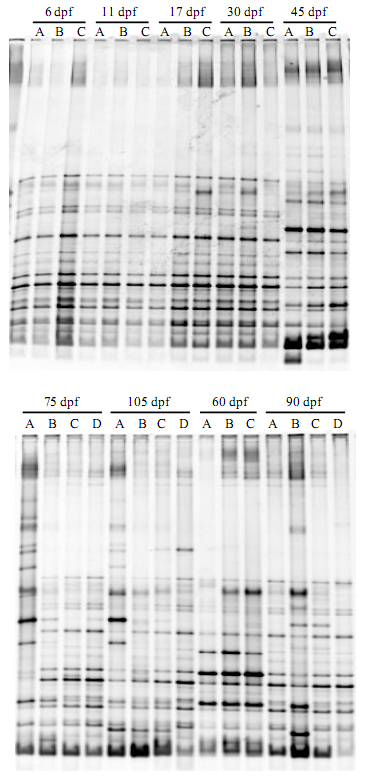

Supplement: Figure S2 — DG-DGGE patterns of the amplified 16S ribosomal RNA (rRNA) gene fragments, each band was considered as an operational taxonomic unit (OTU). For each sample code, the number represents days post-fertilisation (dpf) and the letter refers to a different zebrafish individual sampled at that dpf (to be continued). (TIF) [file pone.0030603.s002.tif]
