# Supplementary material for: Bacterial Community Assembly and Turnover within the Intestines of Developing Zebrafish
Source: PLoS One. 2012 Jan 19;7(1):e30603. doi: 10.1371/journal.pone.0030603 (PMC3261916; doi:10.1371/journal.pone.0030603)
Supplement: Table S2 — General linear models and summary statistics. (DOC) [file pone.0030603.s004.doc]

**Table S2.**  General linear models (GLM) and summary statistics.

| Model: Taxa Richness (S) versus Time, Habitat size and Food diet | | | | | | |  |
| --- | --- | --- | --- | --- | --- | --- | --- |
| Source | DF | | Seq SS | Adj SS | Adj MS | F | P |
| Time (dpf) | 1 | | 68.999 | 41.814 | 41.814 | 9.08 | 0.006 |
| Habitat size (nL) | 1 | | 1.028 | 1.21 | 1.21 | 0.26 | 0.613 |
| Food diet | 1 | | 4.052 | 4.052 | 4.052 | 0.88 | 0.357 |
| Error | 26 | | 119.788 | 119.788 | 4.607 |  |  |
| Total | 29 | | 193.867 |  |  |  |  |
|  |  | |  |  |  |  |  |
|  | S = | | 2.14645 | R-Sq = | 38.21% | R-Sq(adj) = | 31.08% |
|  | |  |  |  |  |  |  |
| Model: Taxa Richness (S) versus Time and Food diet | | | | | |  |  |
| Source | DF | | Seq SS | Adj SS | Adj MS | F | P |
| Time (dpf) | 1 | | 68.999 | 48.866 | 48.866 | 10.9 | 0.003 |
| Food diet | 1 | | 3.87 | 3.87 | 3.87 | 0.86 | 0.361 |
| Error | 27 | | 120.998 | 120.998 | 4.481 |  |  |
| Total | 29 | | 193.867 |  |  |  |  |
|  |  | |  |  |  |  |  |
|  | S = | | 2.11693 | R-Sq = | 37.59% | R-Sq(adj)= | 32.96% |
|  | |  |  |  |  |  |  |
| Model: Taxa Richness (S) versus Habitat size and Food diet | | | | | | |  |
| Source | DF | | Seq SS | Adj SS | Adj MS | F | P |
| Habitat size (nL) | 1 | | 23.943 | 8.262 | 8.262 | 1.38 | 0.25 |
| Food diet | 1 | | 8.322 | 8.322 | 8.322 | 1.39 | 0.249 |
| Error | 27 | | 161.602 | 161.602 | 5.985 |  |  |
| Total | 29 | | 193.867 |  |  |  |  |
|  |  | |  |  |  |  |  |
|  | S = | | 2.44648 | R-Sq = | 16.64% | R-sq(adj) = | 10.47% |
|  | |  |  |  |  |  |  |
| Model: Taxa Richness (S) versus Habitat size and Time | | | | | |  |  |
| Source | DF | | Seq SS | Adj SS | Adj MS | F | P |
| Habitat size (nL) | 1 | | 23.943 | 1.028 | 1.028 | 0.22 | 0.64 |
| Time (dpf) | 1 | | 46.083 | 46.083 | 46.083 | 10.05 | 0.004 |
| Error | 27 | | 123.84 | 123.84 | 4.587 |  |  |
| Total | 29 | | 193.867 |  |  |  |  |
|  |  | |  |  |  |  |  |
|  | S = | | 2.14165 | R-Sq = | 36.12% | R-Sq(adj) = | 31.39% |
|  | |  |  |  |  |  |  |
| Model: Taxa Richness (S) versus Time | | | |  |  |  |  |
| Source | DF | | Seq SS | Adj SS | Adj MS | F | P |
| Time (dpf) | 8 | | 121.533 | 121.533 | 15.192 | 4.41 | 0.003 |
| Error | 21 | | 72.333 | 72.333 | 3.444 |  |  |
| Total | 29 | | 193.867 |  |  |  |  |
|  |  | |  |  |  |  |  |
|  | S = | | 1.85592 | R-Sq = | 62.69% | R-Sq(adj)= | 48.48% |
