# Supplementary material for: Bacterial Community Assembly and Turnover within the Intestines of Developing Zebrafish
Source: PLoS One. 2012 Jan 19;7(1):e30603. doi: 10.1371/journal.pone.0030603 (PMC3261916; doi:10.1371/journal.pone.0030603)
Supplement: Text S1 — Intestine removal methods. (DOC) [file pone.0030603.s005.doc]

**Text S1.** Intestine removal methods.

All procedures were performed using sterile instruments under dissection stereomicroscope according Rawls et al. (2004) and Pham et al. (2008). To be more specific, all surgical instruments and microscope slides used herein were sterilized in autoclave sterilizer for at least 15 minutes, and then washed with 70% ethanol-cotton. The euthanized fish was also wiped with 70% ethanol-cotton, then using the sterilized instruments to separate and collect the intestine under a sterile environment. The separated intestine was wiped with 70% ethanol-cotton before removed to sterile centrifuge tube (1.5 mL) for following DNA extraction.

**References**

Rawls JF, Samuel BS, Gordon JI (2004) Gnotobiotic zebrafish reveal evolutionarily conserved responses to the gut microbiota. Proc Natl Acad Sci USA 101: 4596-4601.

Pham LN, Kanther M, Semova I, Rawls JF (2008) Methods for generating and colonizing gnotobiotic zebrafish. Nat Protoc 3: 1862-1875.
